# Rural-Urban Differences in Social Isolation and Dementia Risk Among Older Adults in India

**DOI:** 10.1093/geroni/igaf122.3120

**Published:** 2025-12-31

**Authors:** Aarushi Vatsyayan, Nasim Ferdows

**Affiliations:** Northeastern University, Boston, Massachusetts, United States; Northeastern University, Boston, Massachusetts, United States

## Abstract

**Background:**

Social isolation is a well-documented risk factor for dementia, but its impact varies by demographic and geographic context. Older adults in India face unique structural and social challenges that may exacerbate dementia risk, particularly in rural areas where healthcare access and support networks are weaker. Understanding these disparities is critical for designing targeted interventions that address region-specific needs.

**Methods:**

This study utilizes data from the Longitudinal Ageing Study in India (LASI) to examine differences in social isolation between rural and urban older adults (ages 45+). We conducted a descriptive analysis to explore variations in social isolation patterns, marital status, family structure, and community engagement. Key variables include demographics, health indicators (Alzheimer’s/dementia history and treatment), family structure, and psychosocial factors (loneliness, depression, and social engagement).

**Results:**

Among 71,513 participants, rural adults exhibited higher rates of social isolation than urban counterparts. Rural older adults were more likely to be married (70.16% vs. 66.49%) but reported lower levels of community engagement. Additionally, rural participants experienced loneliness more frequently, with 22.08% reporting feeling lonely sometimes (vs. 20.04% urban) and 3.79% feeling lonely most/all of the time (vs. 3.51% urban).

**Conclusion:**

Older adults in rural India experience higher social isolation due to limited social and healthcare resources. This study highlights the need for community-based programs, strengthened family and social networks, and improved access to mental health support in rural settings.

